# Low-Resolution Structure of the Full-Length Barley (*Hordeum vulgare*) SGT1 Protein in Solution, Obtained Using Small-Angle X-Ray Scattering

**DOI:** 10.1371/journal.pone.0093313

**Published:** 2014-04-08

**Authors:** Michał Taube, Joanna R. Pieńkowska, Artur Jarmołowski, Maciej Kozak

**Affiliations:** 1 Department of Macromolecular Physics, Faculty of Physics, Adam Mickiewicz University, Poznań, Poland; 2 Department of Cell Biology, Institute of Experimental BiFology, Faculty of Biology, Adam Mickiewicz University, Poznań, Poland; 3 Department of Gene Expression, Institute of Molecular Biology and Biotechnology, Faculty of Biology, Adam Mickiewicz University, Poznań, Poland; Weizmann Institute of Science, Israel

## Abstract

SGT1 is an evolutionarily conserved eukaryotic protein involved in many important cellular processes. In plants, SGT1 is involved in resistance to disease. In a low ionic strength environment, the SGT1 protein tends to form dimers. The protein consists of three structurally independent domains (the tetratricopeptide repeats domain (TPR), the CHORD- and SGT1-containing domain (CS), and the SGT1-specific domain (SGS)), and two less conserved variable regions (VR1 and VR2). In the present study, we provide the low-resolution structure of the barley (*Hordeum vulgare*) SGT1 protein in solution and its dimer/monomer equilibrium using small-angle scattering of synchrotron radiation, *ab-initio* modeling and circular dichroism spectroscopy. The multivariate curve resolution least-square method (MCR-ALS) was applied to separate the scattering data of the monomeric and dimeric species from a complex mixture. The models of the barley SGT1 dimer and monomer were formulated using rigid body modeling with *ab-initio* structure prediction. Both oligomeric forms of barley SGT1 have elongated shapes with unfolded inter-domain regions. Circular dichroism spectroscopy confirmed that the barley SGT1 protein had a modular architecture, with an α-helical TPR domain, a β-sheet sandwich CS domain, and a disordered SGS domain separated by VR1 and VR2 regions. Using molecular docking and *ab-initio* protein structure prediction, a model of dimerization of the TPR domains was proposed.

## Introduction

**Figure 1:**
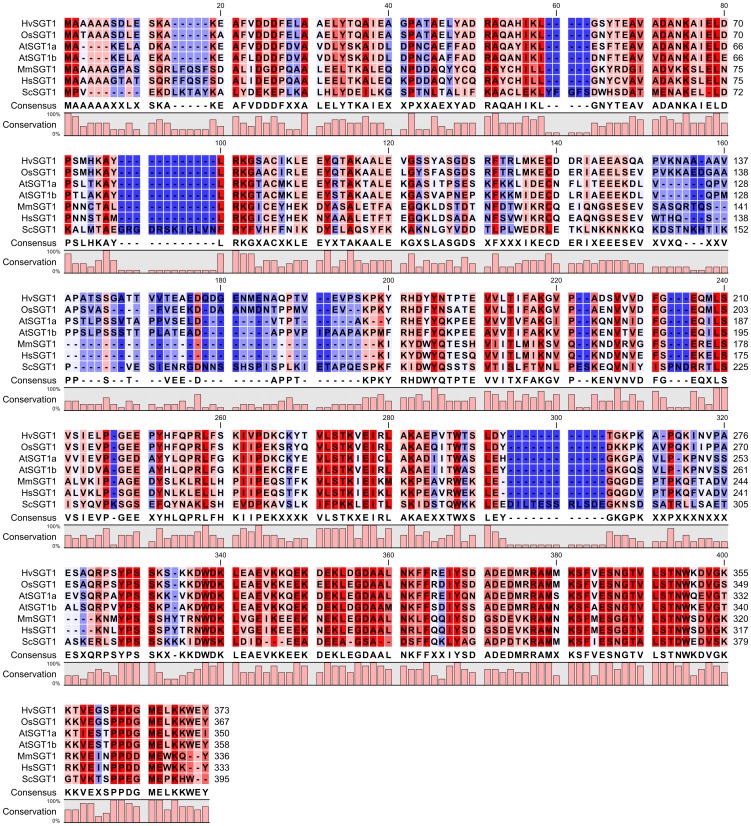
Alignment of SGT1 proteins. *Hordeum vulgare* (HvSgt1), *Oryza sativa* (OsSgt1), *Arabidopsis thaliana* (AtSgt1a and AtSgt1b – isoforms a and b respectively), *Mus musculus* (MmSgt1), *Homo sapiens* (HsSgt1), *Saccharomyces cerevisiae* (ScSgt1).

SGT1, a suppressor of the G2 allele of skp1, is a highly conserved (Figure 1) and essential protein found in all eukaryotic organisms [1]. The SGT1 protein was originally discovered as a suppressor of the skp1-4 mutant in *Saccharomyces cerevisiae*, causing defects in kinetochore assembly, and also as a protein that interacts with the SCF ubiquitin ligase complex [2]. Moreover, the yeast SGT1 protein is also important for the activation of the adenylyl cyclase protein Cdc35p. The human SGT1 protein is involved in not only kinetochore assembly, but also innate immunity [3], [4]. The human nucleotide-binding domain- and leucine-rich repeat-containing proteins (NB-LRR) NOD1 and NLRP3 require SGT1 for proper functioning in response to bacterial peptidoglycan derivatives [4], [5]. In plants, SGT1 is essential for disease resistance mediated by numerous NLR proteins [6]. Mutation or silencing of *SGT1* increases the susceptibility of plants to pathogen attack and growth. Plant SGT1 is also involved in the auxin and jasmonate response, which is mediated by SCF-ubiquitin ligase complexes [7].

**Figure 2:**
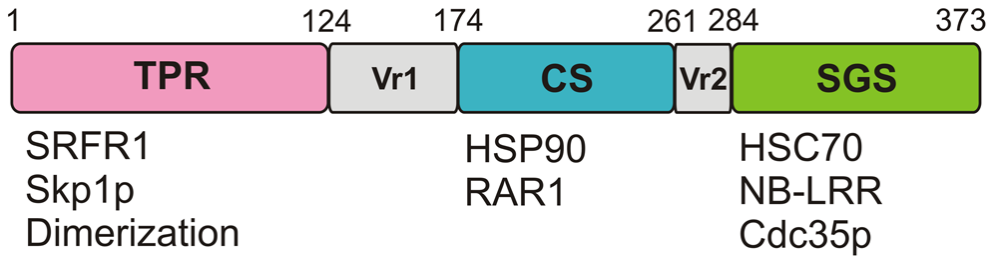
Schematic representation of the SGT1 protein structure. The tetratricopeptide repeats domain (TPR), the CHORD- and SGT1-containing domain (CS), the SGT1-specific domain (SGS), and two less conserved variable regions (VR1 and VR2). Top: position in the sequence of the full-length barley SGT1 protein. Bottom: processes involving a given SGT1 domain or proteins with which the domain interacts.

The SGT1 protein consists of three structurally independent domains (the tetratricopeptide repeats domain (TPR), the CHORD- and SGT1-containing domain (CS), and the SGT1-specific domain (SGS)), and two less conserved variable regions (VR1 and VR2), which lie between the conserved domains (Figure 2) [8]. So far, the best-characterized domain of SGT1 is the CS domain, which has significant structural homology to the yeast HSP90 (heat shock protein 90) co-chaperone p23 [9]–[11]. Indeed, the CS domain of plant SGT1 interacts with the N-terminal nucleotide-binding domain of the HSP90 protein, but in a manner different from the binding of the p23 protein to HSP90 in yeast. The interaction of SGT1 with HSP90 is essential for disease resistance in plants, and mutations in the binding interface result in disease susceptibility [9]. The CS domain of plant SGT1s is also involved in interaction with RAR1 (required for Mla12 resistance), another factor that plays a significant role in innate immunity [8]. SGT1, together with RAR1 and HSP90, forms a multimeric complex that mediates disease resistance, most likely by maintaining appropriate folding and stability of the plant NB-LRR receptors [12]. The SGS domain interacts with the leucine-rich repeat domains (LRR) of NB-LRR resistance proteins in plants and humans, and also with the LRR domain of the yeast cdc35p adenylyl cyclase [4], [13], [14]. Phosphorylation of Ser361 in the SGS domain of yeast SGT1 by CK2 prevents dimerization and kinetochore assembly [15]. The SGS domain is also crucial for the auxin response that is mediated by SGT1 in *Arabidopsis thaliana* [7]. However, there is very limited information about the role of the TPR domain of SGT1 in plants. In *Arabidopsis thaliana*, the expression of two SGT1 isoforms, AtSGT1a and AtSGT1b, depends on the TPR domain sequence [16]. Recently, it has been shown that AtSGT1a and AtSGT1b interact with the SRFR1 (suppressor of rps4-RLD1) protein via their TPR domains, although the functional role of this interaction is still unknown [17]. In yeast, the TPR domain of SGT1 is required for kinetochore assembly and also for binding to the Skp1 protein [18]. Surprisingly, the TPR domain of SGT1 does not bind to HSP90, unlike many other HSP90 interacting proteins that contain TPR domains [11], [12].

It has recently been observed that plant and yeast SGT1 proteins can dimerize in a low ionic strength environment and at high protein concentrations [18], [19]. This dimerization process is functionally important in yeast, where a mutation that inhibits the dimerization of SGT1 causes problems in kinetochore assembly. Moreover, a TPR deletion mutant of SGT1 that contains the dimerization module of the yeast CENP-B protein is functional in kinetochore assembly [18]. One study identified loss-of-function and gain-of-function mutations in *SGT1* by testing the susceptibility of wild-type *Nicotiana benthamiana* plants for to PVX virus infection and discovered a dominant-negative mutation in AtSGT1b (Glu119Gly) [9]. The mutated residue is located in the C-terminal helix of the TPR domain, and may play a role in dimerization, although this remains to be verified. Dimerization is also inhibited by the oxidation of cysteine residues in the TPR domain [19]. Dimerization of the TPR domains may regulate the response to stress because ionic strength and redox state can change the oligomerization status of SGT1. In contrast to plant and yeast SGT1s, the human SGT1 protein does not undergo dimerization, most likely because of the lack of polar residues in the C-terminal capping helix of the TPR domain [19].

Small-angle X-ray scattering (SAXS) is one of the most effective experimental methods for analysis of protein structure in solution. This method is especially useful for proteins that are difficult to crystallize (e.g., multidomain proteins with flexible regions, multimeric complexes or intrinsically disordered proteins) (for review see: [20]–[22]). Therefore, the SAXS method can be used to produce reliable results for molecules with a wide range of sizes, including not only proteins [23]–[25] but also macromolecular complexes, including nanosystems such as viruses [26] or ribosomes [27]. Recently, a few methods have been proposed for the determination of the low-resolution structure of proteins in solution on the basis of synchrotron SAXS data [20], [28], [29].

There is limited structural information on full-length SGT1 proteins. In this study, we used SAXS and synchrotron radiation to determine, for the first time, the structure and properties of the full-length SGT1 protein from barley (*Hordeum vulgare*). In solution, the plant SGT1 protein exists in monomer-dimer equilibrium; with increasing ionic strength, this equilibrium is shifted towards the monomeric state. Using a novel SAXS data analysis method [30], [31] (including singular value decomposition and multivariate curve resolution alternating least-squares procedures), we were able to extract the pure scattering function of the monomeric and dimeric forms and to create low-resolution models using *ab-initio* methods.

## Experimental

### Protein expression and purification

#### Full length barley SGT1

The full length barley SGT1 cDNA cloned into the pET151-D TOPO (Invitrogen, Carlsbad, CA, USA) vector was used for the transformation of *Escherichia coli* BL21 DE3 Codon Plus. The cells were cultured in Luria Broth (LB) liquid medium supplemented with ampicillin (50 µg/ml) and chloramphenicol (30 µg/ml) (final concentrations), and a total of 1.6 L of LB medium was inoculated with 40 ml of overnight culture. The culture was incubated at 37°C with shaking (200 rpm) until the OD^600 nm^ reached 0.6–0.8; IPTG was then added at a final concentration of 1 mM. Cells were grown for 5 hours and harvested by centrifugation at 10000 g at 4°C for 30 minutes and were subsequently frozen at −20°C until further use. The frozen cells were than resuspended at 4°C in lysis buffer (20 mM sodium phosphate pH 7.5, 500 mM NaCl, 30 mM imidazole, 1% Triton X-100, 10 mM 2-mercaptoethanol and EDTA-free protease inhibitor cocktail), disrupted by sonication (5 times 60 sec. on ice) and centrifuged at 10000 g. In the next step, the supernatant was applied to a HisTrap 1 ml FF Ni-NTA column (GE Healthcare Life Sciences, Uppsala, Sweden) using Akta Explorer X10. The column was washed with a binding buffer (20 mM sodium phosphate pH 7.5, 500 mM NaCl, 10 mM 2-mercaptoethanol and 30 mM imidazole), and the barley SGT1 protein with a His-TEV site was eluted using a gradient of elution buffer (20 mM sodium phosphate pH 7.5, 500 mM NaCl, 10 mM 2-mercaptoethanol and 500 mM imidazole). The fractions that contained the barley SGT1 were concentrated using the Amicon 10 kDa MWCO (Millipore) centrifugal filter device and purified using Superdex 200 pg HiLoad 16/60 (GE Healthcare Life Sciences). The first fraction of the SGT1 peak was cleaved using His-Tev (Ser219Val) [32] and the cleaved protein was then purified using HisTrap 1 ml FF. For the final purification step, barley SGT1 was loaded into a MonoQ HR 5/5 (GE Healthcare Life Sciences, Uppsala, Sweden) ion exchange column.

#### TPR domain from *Hordeum vulgare* SGT1

The cDNA sequence of TPR domain from *Hordeum vulgare* SGT1 (6–124) was amplified from the pET151-HvSgt1 plasmid by standard PCR reaction and cloned into the pETM11 vector [33] using NcoI and EcoRI restriction enzyme sites. The protein was expressed in *E.coli* BL21(DE3) Codon Plus cells. Transformed cells were grown at 37°C (to OD^600 nm^ = 0.6–0.8) and induced with 1 mM IPTG. Induced cells were grown overnight at 20°C. After expression, bacterial cells were centrifuged at 4°C at 5500 g for 30 minutes and frozen at −20°C. For purification, the cells were thawed on ice and resuspended in buffer A (50 mM sodium phosphate pH 7.5, 500 mM NaCl, 30 mM imidazole and 10 mM 2-mercaptoethanol) supplemented with 0.1% Triton X and protease inhibitor cocktail. The cells were disrupted by five cycles of sonication (30 sec. on/1 minute on ice) and centrifuged for 1 hour at 10000 g at 4°C. The cleared lysate was loaded onto the HisTrap FF 1 ml column washed with 10CV of buffer A and eluted with gradient 0–100% of buffer B (50 mM sodium phosphate pH 7.5 500 mM NaCl 500 mM imidazole and 10 mM 2-mercaptoethanol) over 20CV. The fractions containing TPR domain protein were collected, supplemented with TEV protease for his-tag cleavage and dialyzed against buffer A overnight at 4°C. The TEV protease and unclaved TPR domain were removed by the HisTrap FF 1 ml column. The TPR protein was concentrated and purified by size exclusion chromatography using HiLoad 16/60 Superdex 200 pg column equilibrated in buffer C (20 mM sodium phosphate pH 7.5, 200 mM NaCl, 10 mM 2-mercaptoethanol). The purified protein was concentrated to 10 mg/ml, snap frozen in liquid nitrogen and stored at −80°C for further use.

#### TPR-CS and CS-SGS domains from *Hordeum vulgare* SGT1

The variants of barley SGT1 protein: TPR-CS (1–261) and CS-SGS (174–373) obtained after the expression in vector pnEA-vH [34] (the same conditions as for TPR domain) were purified by affinity chromatography on a column HisTrap FF 1 ml and by gel filtration on a Superdex 200 pg HiLoad 16/60 column, similarly as TPR domain. The samples were concentrated to 14 mg/ml and 15 mg/ml in a buffer 20 mM Tris pH 7.5 150 mM NaCl, 10 mM 2-mercaptoethanol. For measurements of circular dichroism the samples were diluted to 1∶10 in a buffer 10 mM NaH_2_PO_4_ pH 7.5, 2 mM 2-mercaptoethanol and dialysed overnight for 12 hours against buffer A.

#### CS domains from *Hordeum vulgare* SGT1and from *Arabidopsis thalina* SGT1a

CS domains from barley SGT1 protein and from *Arabidopsis thalina* SGT1a protein were cloned into vectors pETM41 [33] and pETM30, respectively. The protein expression was performed in the same conditions as that of TPR domain. The proteins were purified similarly as the other variants of SGT1 protein by passing through a bed of Ni-NTA agarose (Pierce) and gel filtration on a Superdex 200 10/300 GL column. The samples for circular dichroism measurements were dialysed into a buffer 20 mM NaH_2_PO_4_ pH 7.5, 5 mM 2-mercaptoethanol.

### Circular dichroism (CD)

The protein samples for CD measurements were dialyzed overnight against a 10-mM sodium phosphate buffer (pH 7.5) at 4°C. Following dialysis, the sample was centrifuged at 14000 rpm for 30 minutes at 4°C and filtered through a 0.1-µm filter. CD measurements were conducted at room temperature using diluted protein samples (c = 10–47 µM) and a Jasco J-815 spectropolarimeter (Jasco, Japan) with a 0.02 cm cell length path. For full-length SGT1 (c = 12 µM) a total of six scans, ranging from 176 nm to 280 nm, were collected. CD data for TPR-CS (c = 10.4 µM), CS-SGS (12.8 µM) were collected from 190 nm to 280 nm. CS domain from barley SGT1 protein (c = 47 µM) and CS domain of SGT1a from *Arabidopsis thalina* (46.7 µM) CS domain form were studied from 205 nm to 280 nm. All CD data were collected at a 50 nm/sec scanning speed and 1 nm band-width. Buffer subtraction and spectra deconvolution procedures were conducted using the Jasco Spectra Manager II software and using CDSSTR module on Dichroweb server [35], [36].

### Structure prediction

The three-dimensional structures of individual SGT1 domains (TPR, CS and SGS) were generated using a computer algorithm for *ab initio* protein folding and structure prediction, QUARK (TPR domain) and iterative structure assembly and refinement approach implemented in I-TASSER (CS and SGS domains) [37]–[39]. The models were obtained on the basis of the barley SGT1 amino-acid sequence and built from small polypeptide moieties (1–20 residues long) by replica-exchange Monte Carlo simulation, using the *ab initio* modeling server QUARK (http://zhanglab.ccmb.med.umich.edu/QUARK/) and using first template search in PDB data base and then replica-exchange Monte Carlo simulations with additional loop modeling with structure refinement in the third step (http://zhanglab.ccmb.med.umich.edu/I-TASSER/).

### Small angle X-ray scattering measurements

#### Full length barley SGT1

SAXS measurements were performed on the X33 Beamline of EMBL Hamburg Outstation on the DORIS storage ring at DESY [40], [41]. Scattering patterns were recorded on a semiconductor photon counting Pilatus 1M-W (Dectris) pixel detector (67×420 mm^2^), with a sample-to-detector distance of 2.7 m, which corresponds to an s-axis range of 0.06 to 6 nm^−1^, using synchrotron radiation (wavelength λ = 0.15 nm). All measurements were performed using a flow cell (70 µl volume) with polycarbonate windows [42] and automated filling at 10°C. The detector s-axis (where s = 4πsinθ/λ with a scattering angle of 2θ) was calibrated using the diffraction patterns of silver behenate [43]. The measurements were carried out on a series of SGT1 solutions in 50 mM Tris/HCl pH 7.5 and 5 mM DTT with salt concentrations of 0, 0.5 or 1 M, in 4 successive 30-s frames. Protein concentrations of 3.46, 3.41 and 2.71 mg/mL in solutions containing 0, 0.5 and 1 M NaCl, respectively, were estimated by the 280 nm absorption method, using a molar extinction coefficient of ε = 44350 M^−1^ cm^−1^. The solution scattering data were corrected for detector response and normalized to the incident beam intensity, and the scattering of the buffer was subtracted using the program package PRIMUS [43]. Solutions of a known concentration (∼3 mg/mL) of bovine serum albumin (BSA) xylose/glucose isomerase from *Streptomyces rubiginosus* were used as references for the molecular weight calibration [44].

#### TPR domain from *Hordeum vulgare* SGT1

Solution scattering data for TPR domain were collected on the BM29 BioSAXS Beamline [45] of ESRF (Grenoble, France) using synchrotron radiation (wavelength λ = 0.9919 nm). SAXS images (10 frames/sample) were recorded on Pilatus 1M 2D detector (active area: 169×179 mm^2^) and with a sample-to-detector distance of 2.867 m, (s-axis range of 0.06 to 6 nm^−1^). For small angle X-ray scattering experiment the TPR domain was dialyzed against low salt buffer (50 mM Tris HCl pH 7.5, 5 mM DTT). The samples were prepared by mixing dialyzed protein with high salt buffer (50 mM Tris HCl pH 7.5, 5 mM DTT, 3 M KCl) for final KCl concentration: 0, 100, 200, 400, 600 oraz 800 mM and protein concentration 2 mg/ml. All experiments were done at 15°C using continuous flow cell (sample volume = 30 µl). SAXS data were processed using the same procedures and software as for full length barley SGT1.

### Singular Value Decomposition (SVD) analysis

To estimate the number of species in solutions of the barley SGT1 protein and TPR domain, we employed singular value decomposition (SVD) analysis using the PRIMUS [43] software and SVD function in MATLAB. Scattering data set matrix A of dimensions I×J represents I scattering curves of J points and can be decomposed into a product of three matrices: U, S, and V (Equation 1).
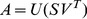
(1)Matrix U represents eigenvectors that could be linearly combined using coefficients from matrix (SV)^T^ to reconstruct the data set A. To minimize errors in the SVD analysis, we used experimental data to 2 nm^−1^. Using PRIMUS, we established that only two eigenvectors were statistically important. The PRIMUS result was also confirmed by analyzing the autocorrelation of the calculated eigenvectors from matrix U, which showed that only two eigenvectors had non-zero autocorrelation functions. Pure scattering function reconstruction of the components was conducted using the Multivariate Curve Resolution Alternating Least-Squares (MCR-ALS) algorithm implemented in MATLAB [30], [31]. In the MCR-ALS algorithm, the data set matrix D (Equation 2) is represented by the sum of the matrix product CS and matrix E, where C is the concentration matrix, S is the matrix of pure scattering functions of species and matrix E represents errors. The dimension of D and E is I×J, where I is the number of scattering curves of J points, and S has dimensions N×J, where N is the number of components in the system. C has dimensions I×N.
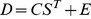
(2)The initial estimation of scattering curves was conducted using evolving factor analysis. In the final estimation, we used non-negativity constraints for scattering function and the requirement of the presence of only two species (sum of the concentration coefficients must be equal to one). The reconstructed pure scattering functions of individual components were used for the analysis and for *ab-initio* SAXS modeling.

### Modeling of the low- resolution structure

Two independent programs, DAMMINv51 [46] and GASBOR v22 [47], were used to determine the low-resolution structures of the monomeric and dimeric forms of full-length barley SGT1 in solution. In total, 15 independent DAMMIN bead models were calculated to check the stability of the solution. The models obtained from DAMMIN were averaged to determine the most typical shapes of the SGT1 monomer and dimer forms, using the programs DAMAVER and SUPCOMB [43]. Another program used for the reconstruction of the low-resolution structure of SGT1 was GASBOR. The procedure for the determination of the structural models in GASBOR is based on a similar strategy as in DAMMIN, but the models are built as a chain-compatible spatial arrangement of the dummy atom residues. The number of dummy residues was equal to the number of C atoms. To estimate the degree of the dynamics and conformational heterogeneity of the monomeric and dimeric forms of SGT1, we analyzed the SAXS data using an ensemble optimization method (EOM) [21]. This method randomly generates a large number of models of multidomain proteins using the rigid body approach. In the next step, the fraction of the models that creates an ensemble with the best fit to the experimental data is selected using a genetic algorithm.

To confirm the barley SGT1 models obtained from *ab initio* modeling, we also applied rigid body modeling, using the program BUNCH [48] from the ATSAS package. SGT1 domains were modeled using the protein structure prediction servers QUARK and I-TASSER. In the rigid body modeling, the flexible regions between the rigid domains were represented as dummy residues, with no structural constraints.

Modelling of the TPR dimer against SAXS data was performed in SASREF 6.0 [48] from ATSAS package (on-line server version) with imposed P1 or P2 symmetry using the structural model of TPR domain generated by Quark and SAXS data (after MCR-ALS analysis). The data set of 15 independent TPR domain dimer models was generated. All models have almost the same structure. Molecular contacts calculated from SASREF model were further used in rigid body modelling program CORAL.

Modeling of the barley SGT1 dimer was performed using the CORAL program from the ATSAS package, and the scattering curve was obtained from the MCR-ALS analysis. CORAL combines two algorithms, rigid body modeling from SASREF and multidomain protein modeling using rigid body and *ab-initio* modeling from BUNCH, to model multiple chain and domain proteins [48]. With the CORAL program, it is possible to model multichain protein complexes without imposing any symmetry. In the CORAL runs, contacts from the SASREF model of the TPR domain dimer were used as an additional constraint, and no symmetry was imposed.

## Results

### Modeling of the TPR, SGS and CS domains of the barley SGT1 protein and its dimerization via TPR domains

The use of *ab-initio* modeling was necessary because there is no structural model of the barley SGT1 protein. The structure of the three domains of the barley SGT1 protein was modeled using the *ab-initio* modeling platform QUARK and meta-threading approach approach implemented in I-TASSER, which were chosen because are ranked as the best platform in the free-modeling and protein prediction competition in CASP9 [49]. The structural model of the TPR (6–124 aa) domain of barley SGT1 is very similar to the known structures of other TPR domain-containing proteins [9], [11] (see Figure S1 and Table S1). The model of the TPR domain has a typical fold consisting of three helix-turn-helix motifs (three tetratricopeptide repeats), with a solvating helix at the C terminus. The model of the CS domain (173–261 aa) is similar to the structure of the CS domains from human and *Arabidopsis* SGT1 homologs and has a typical beta sandwich structure (see Figure S2). The structure of the barley SGS domain (284–371 aa) consists of a three-helix bundle and regions with no secondary structures at the C terminus; this structure corroborates NMR spectroscopic results showing only a limited α-helical secondary structure content in the NMR spectrum of the isolated human SGS domain [11].

**Figure 3:**
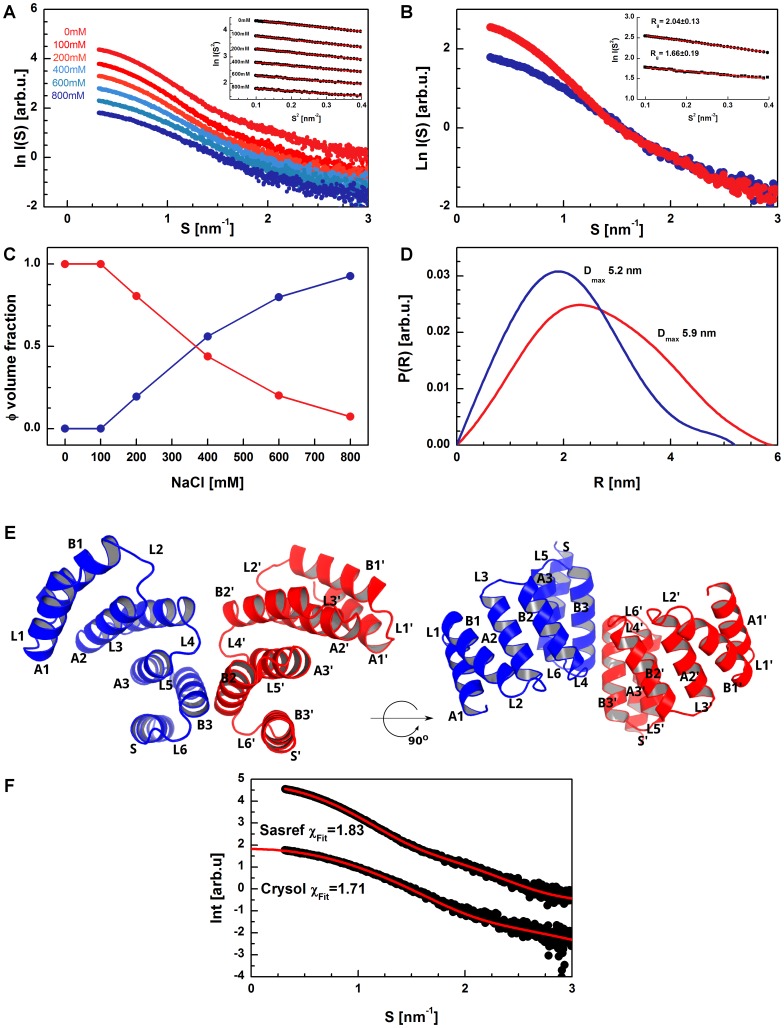
The structure of the TPR dimer. Scattering profiles of the TPR domain from barley SGT1 protein in solution at different salt concentrations (A) and the Guinier plot of scattering curves (inset). Scattering profiles of the TPR monomer (blue) and dimer (red) obtained using the MCR-ALS procedure (B) and the Guinier plot of scattering curves (inset). Volume fractions of TPR monomer (blue) and dimer (red) (C). Normalized pair distance distribution functions p(r) and maximum diameter of particles computed using GNOM (D). The structure of the TPR dimer, predicted using the SASREF and SAXS data (E). Helices denoted as A and B are first and second in the tetratricopeptide repeat, and S is the solvating helix at the C terminus. The loops (L) between helices are numbered in the order they appear, from the N to the C terminus. Direct comparison of theoretical scattering curves for TPR dimer and monomer with experimental SAXS data (F).

In the first step of the data analysis, a three-dimensional model of the barley SGT1 TPR domain (6–124 aa) was obtained using the online modeling platform QUARK (http://zhanglab.ccmb.med.umich.edu/QUARK/). The model has a canonical TPR structure with three tetratricopeptide repeats, each repeat being formed by two anti-parallel helices. A solvating helix is located at the C terminus of the domain. This helix is typical for all structures of TPR domains solved so far, and it is hypothesized to increase the solubility and stability of the TPR fold [50], [51]. In our model, two cysteine residues are located very close to each other, which is in good agreement with the observation that cysteine oxidation disrupts dimer formation [19]. Because little is known about the dimerization interface between the TPR domains, the structure of the TPR homodimer was predicted using the SAXS data and SASREF 6.0 from ATSAS package (on-line server version) [48]. In Figure 3A the experimental SAXS data for TPR domain at different salt concentrations (0–800 mM KCl) are shown. The scattering data after the MCR-ALS analysis for monomeric and dimeric forms of TPR domain are presented in Figure 3B. The radii of gyration (R_G_) characterizing the TPR domain in solution were 1.66 nm and 2.04 nm for TPR monomer and dimer, respectively. In Figure 3C the volume fractions of TPR monomer and dimer at different ionic strength are presented. The observed changes of monomer/dimer volume fractions are characteristic for ionic strength-dependent dimerization. The maximum particle diameters (D_max_) characterizing the TPR dimer and monomer were estimated from the p(r) functions (Figure 3D) and were found to be 5.2 nm and 5.9 nm for the monomer and the dimer, respectively. The predicted dimer has a open topology, as shown in Figure 3E, and is distinct from the crystal structure of the CTPR3Y3-engineered TPR domain, which forms open super-helical oligomers [52]. The interface between TPR domains are formed only by residues from B3 and B3′ helices. In Figure 3F are also presented the results of direct comparison of theoretical scattering curves for TPR dimer and monomer with experimental SAXS data. A high ionic strength environment can influence these interactions and prevent SGT1 dimer formation, which substantiates our SAXS measurements, as well as the results of the Kleanthous and Shirasu laboratories [19].

### Expression and purification of the full-length SGT1 protein from barley

**Figure 4:**
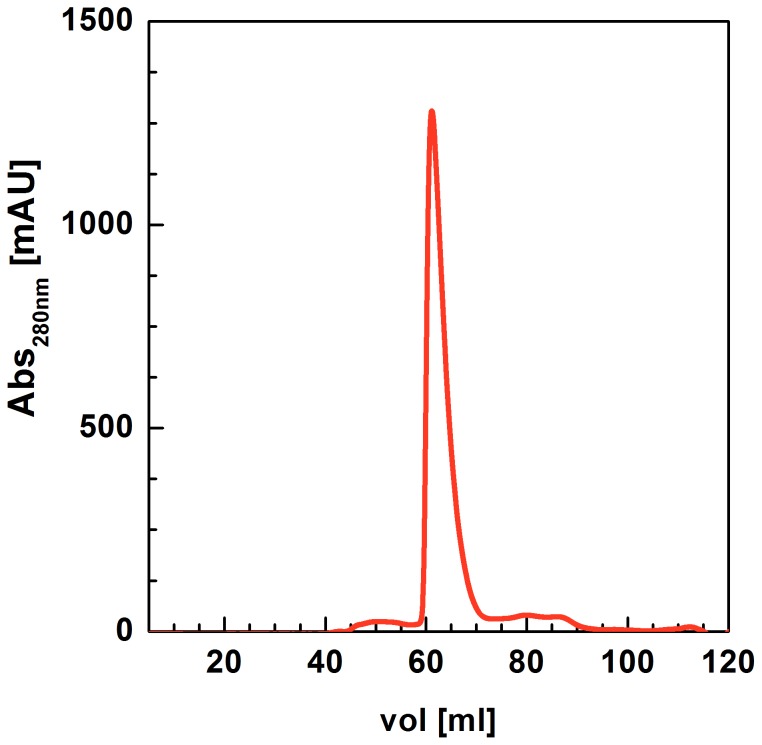
The elution profile of the recombinant full-length barley SGT1 protein purified on a Superdex column.

**Figure 5:**
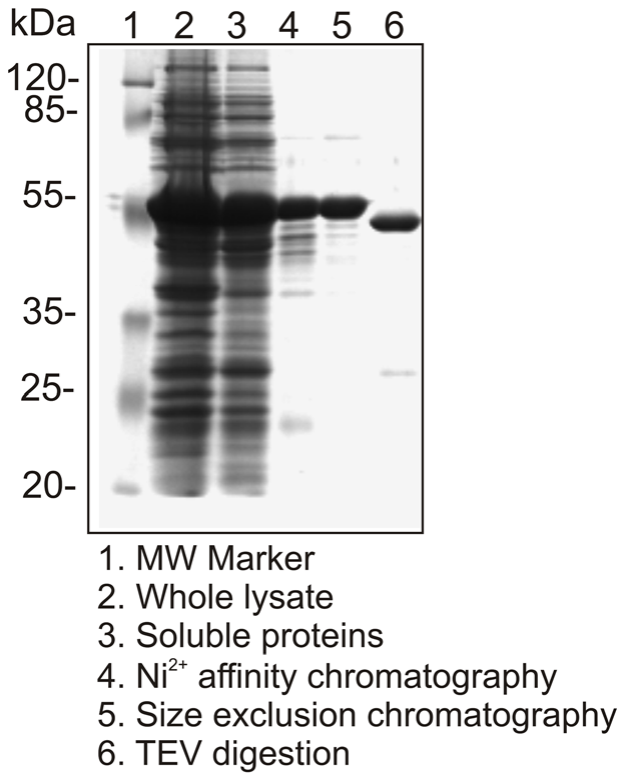
SDS-PAGE analysis of extracts from all of the steps performed during purification of the full-length barley SGT1 protein.

In many protocols describing the expression and purification of SGT1s from various sources [19], degradation issues associated with the recombinant protein purification procedure were emphasized. This degradation occurs regardless of the expression system used (bacteria or insect cells). In this study, we decided to use the prokaryotic expression system, but, to avoid degradation problems, we applied additional purification steps. The elution profile of the recombinant barley SGT1 protein purified on a Superdex column shows an asymmetric peak (Figure 4). Only the first two fractions contained non-degraded SGT1; therefore, only these fractions of the SGT1 peak were cleaved using His-Tev protease (Ser219Val) [32]. After an overnight incubation with the Tev protease, additional degradation was not observed. All the purification steps are summarized in Figure 5 (SDS-PAGE analysis). We ultimately obtained approximately 1 mg of pure protein from 1 L of bacterial culture.

### Circular dichroism studies of the full-length barley SGT1 protein, TPR-CS, CS-SGS, CS domains from barley SGT1 and CS domain from *Arabidopsis thalina* SGT1a

**Figure 6:**
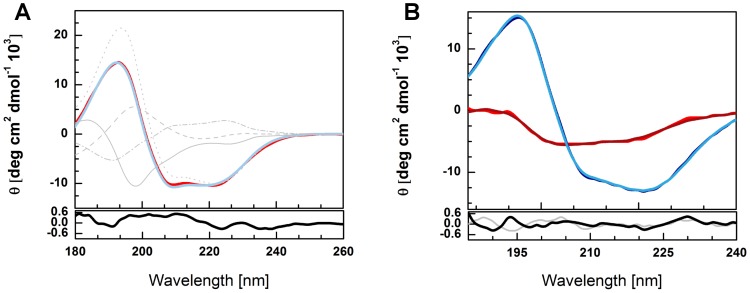
Circular dichroism spectroscopy. CD spectrum (red) and estimated secondary structure of the full-length barley SGT1 protein (blue). Deconvoluted secondary structure elements – α-helix (dots), β-sheet (dash line), turns (dash-dot), and others (solid line). Bottom panel – residuals (A). The CD spectra collected for TPR-CS (blue) and CS-SGS (red) fragments together with their deconvolution (B).

To support the results of our structural studies, the secondary structure content was estimated using circular dichroism spectroscopy. A representative CD spectrum of the barley SGT1 is presented in Figure 6. Two minima at 208 nm and 222 nm, which are characteristic of α-helical proteins, are clearly visible on the CD spectrum. The second minimum has a lower value, which suggests an influence of other secondary structural motifs. An examination of the spectrum, together with its deconvolution, provided us with an estimate of the secondary structure content of the barley SGT1 (Figure 6).

The CD spectra (from 190 nm to 280 nm) collected for TPR-CS and CS-SGS proteins together with their deconvolution are presented also in Figure 6. The experimental CD data for CS domain from barley SGT1 protein and CS domain of SGT1a from *Arabidopsis thalina* (reference protein), as well as CD spectrum for CS-SGS protein (obtained in the presence of 30% Ficoll 70) are presented in Figure S3.

Circular dichroism spectroscopy clearly shows the modular architecture of the barley SGT1 protein, with the α-helical TPR domain, the β-sheet sandwich CS domain, and the disordered SGS domain separated by the VR1 and VR2 regions. The α-helix content of the barley SGT1 protein is 33.9%, which corresponds to the TPR domain. The CS domain of the studied SGT1 is responsible for 21.9% of the β-sheet secondary structure. The SGT1 also has 11.8% of the β-turns and 33.5% of the random coil structure. VR1 and VR2 represent 67 of the total 373 amino acids (18%). Assuming that both VR1 and VR2 are in a random coil conformation, almost 12% of the random coil content must originate from the rest of the protein.

The β-turn content (12%) can be ascribed to some unstructured regions outside the VR1 and VR2 regions. The SGS domain consists of 84 amino acids. Because the isolated SGS domain shows NMR spectra characteristic of unfolded proteins [11], the remaining 12% of the random coil regions may be linked to the unfolded part of the SGS domain.

The content of α-helices determined for TPR-CS protein from CD data was 36% (found mainly in TPR domain), while the content of β-strands was 17% (mainly in CS domain) (see secondary structures of TPR and CS domains presented in Figures S1 and S2). The amount of β-turns can be estimated as 16% and 30% can be ascribed to some unstructured regions of TPR-CS protein. For CS-SGS protein the content of α-helices was only 5%, 29% corresponded to β-strands, and 18% to β-turns. As much as 48% of the CS-SGS protein is totally disordered. Taking into account that the TPR-CS and CS-SGS proteins are smaller than full-length barley SGT1, the above contents of structural elements well correlate with CD results obtained for SGT1.The attempts at determination of the effect of crowded environment (in 30% Ficoll 70) on the CS-SGS protein structuralisation have shown an increase in the content of elements of secondary structure (see Figure S3).

### Direct analysis of the SAXS data of the barley SGT1 protein

**Figure 7:**
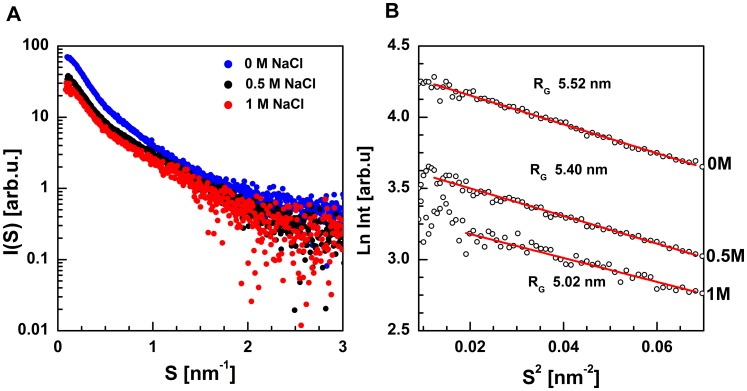
SAXS data. Scattering profiles of the full-length barley SGT1 protein in solution at different salt concentrations (A) and the Guinier plot of scattering curves (B).

To study dimerization of the full-length barley SGT1, we conducted SAXS experiments at various salt concentrations. The solution scattering data of the full-length barley SGT1 protein at various ionic strengths are presented in Figure 7A. With increasing ionic strength, we observed a decrease in the forward scattering intensity I(0), as shown in Figure 7B. Additionally, at low salt concentrations, the barley SGT1 protein forms dimers that fall apart with an increase in the ionic strength of the environment. This behavior is expected for ionic strength-dependent dimerization.

We assumed that the barley SGT1 protein underwent dimerization but not aggregation because the scattering data fitted into the linear range of the Guinier plot, as shown in Figure 7B. Previous studies have demonstrated that the SGT1 protein can dimerize via interactions of the TPR domains [18], [19]. The values of the radii of gyration (R_G_) that characterize the SGT1 protein were found to increase with decreasing ionic strength, suggesting that in solution, SGT1 exists in equilibrium between the monomeric and dimeric states. It indicates that the dimerization process takes place at low ionic strength conditions. The protein sample at the high salt concentration (1 M NaCl) was characterized by the radius of gyration R_G_ = 5.02 nm, and the value of R_G_ increased to 5.52 nm in a low ionic strength environment (0 M NaCl).

**Figure 8:**
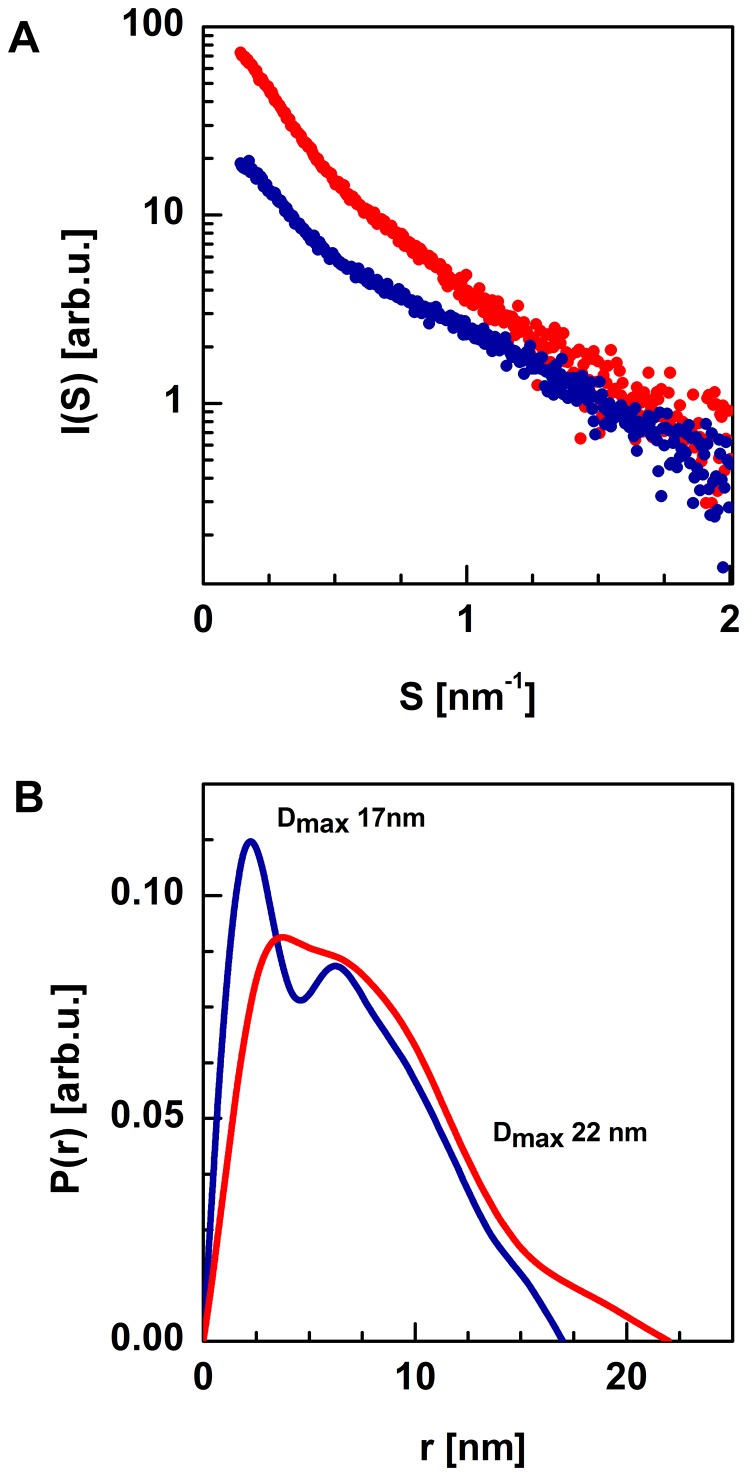
Scattering profiles of the full-length barley SGT1 monomer (blue) and dimer (red) obtained using the MCR-ALS procedure (A). Normalized pair distance distribution functions p(r) and maximum diameter of particle computed using GNOM (B).

As noted previously, a three-dimensional structure of the full-length SGT1 protein has not yet been proposed. Only the structures of the CS domains of the SGT1 protein of *Arabidopsis thaliana* and *Homo sapiens* have been solved, using NMR spectroscopy and X-ray diffraction methods [9]–[11]. To determine the structures of the full-length barley SGT1 monomer and dimer, SAXS data were collected. Unfortunately, the barley SGT1 protein exists in solution in monomer/dimer equilibrium, and raw experimental SAXS data are not adequate to model the SGT1structure. Recently, Blobel and co-workers showed that using a chemometric approach called the multivariate curve resolution alternating least-squares method (MCR-ALS), it is possible to extract pure scattering curves of components from a complex mixture and to use them for low-resolution modeling [28]. This method is ideal for studying a transient protein-protein interaction (to separate the data for two or more oligomeric forms), as in the case of the barley SGT1 protein. For the singular value decomposition (SVD) and MCR-ALS analyses, we used scattering data up to s = 2 nm^−1^ (SAXS data with low S/N ratio beyond 2 nm^−1^ are unsuitable for this type of analysis). This range of SAXS data can be used for shape determination methods and rigid body modeling. In the first stage, using the SVD method [53], it was possible to estimate the number of species in solution, and we discovered only two components (monomer and dimer of the barley SGT1 protein). They too agree well with the biophysical data [19], which also pointed to a monomer/dimer equilibrium of SGT1. The evolving factor analysis (EFA) was chosen for the estimation of scattering curves for a pure monomer and a pure dimer, as well as concentration profiles, prior to refinement [30]. During the refinement process, non-negativity of scattering curves and summation of concentrations to one was assumed. After this procedure, the pure scattering curves for monomeric and dimeric SGT1 forms were obtained. On the basis of the data for the monomeric and dimeric barley SGT1, the intra-particle pair distance distribution functions p(r) were calculated (Figure 8). The maximum particle diameters (D_max_) characterizing the barley SGT1 dimer and monomer were estimated from the p(r) functions and were found to be 17 nm and 22 nm for the monomer and the dimer, respectively. The pair distance distribution function of the monomeric barley SGT1 has two maxima that are characteristic of a multidomain particle. For the barley SGT1 dimer, the first maximum is slightly shifted towards greater distances, and the second maximum is much broader; both peaks are less resolved, a consequence of the dimerization process. The calculated p(r) functions for the monomer and dimer have their maxima in the same positions as the maxima of the functions calculated using raw experimental data at various salt concentrations (data not shown), confirming that the MCR-ALS analysis is accurate.

### *Ab initio* modeling of a low-resolution structure of the barley SGT1 protein

**Figure 9:**
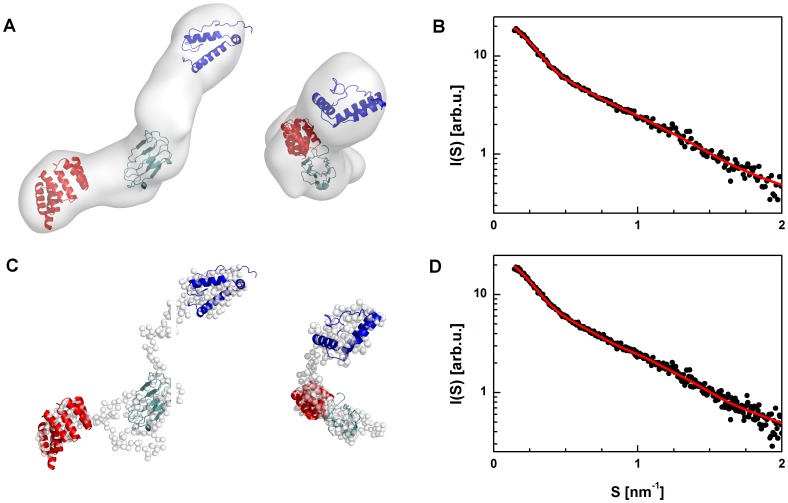
Shape determination of the full-length barley SGT1 monomer. Models of SGT1 domains superimposed on the averaged low-resolution model obtained using DAMMIN (A) and fit of the theoretical scattering curve of the model to MCR-ALS scattering data for the monomer (B). Models of the SGT1 domains superimposed on a low-resolution model obtained by GASBOR (C) and fit of theoretical scattering curve (red) of the model to MCR-ALS scattering data for the monomer (D).

**Figure 10:**
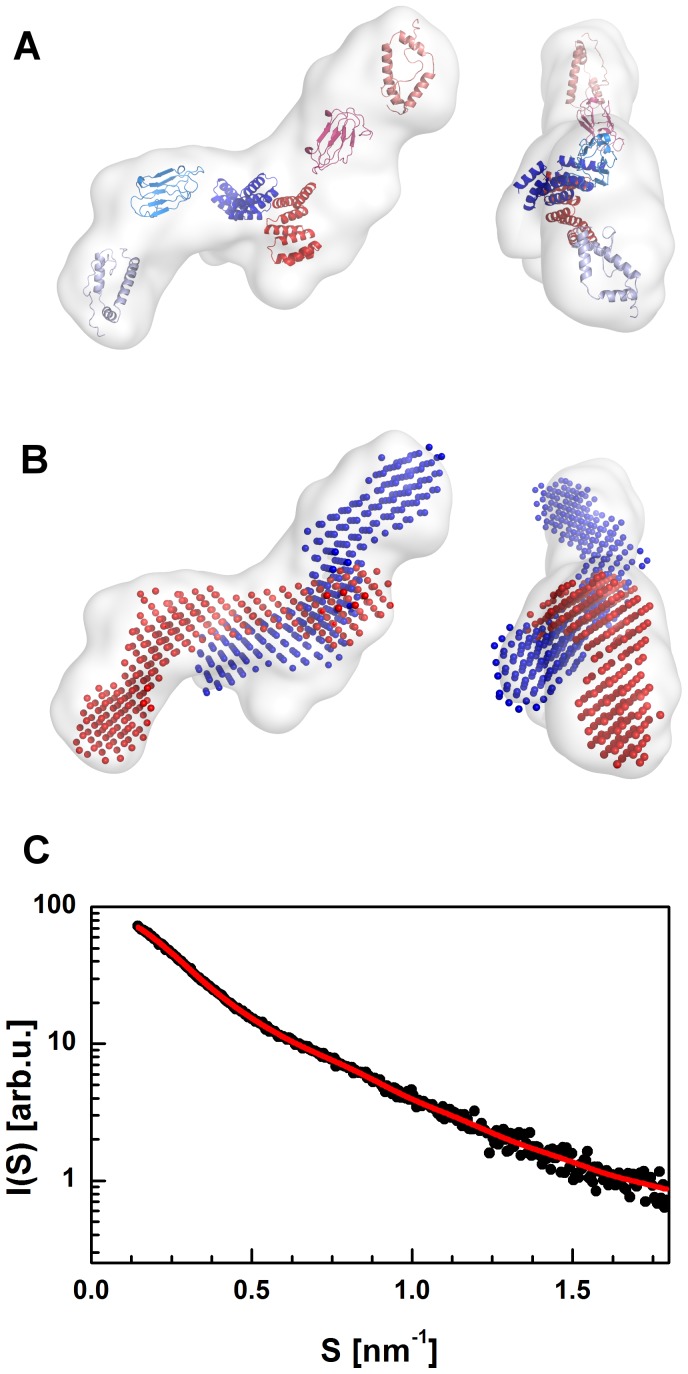
Results of *ab-initio* shape determination of the full-length barley SGT1 dimer. Models of SGT1 domains superimposed on the low-resolution model obtained by DAMMIN (A). The low-resolution models (red and blue) of SGT1 monomers superimposed on the low-resolution model of the SGT1 dimer obtained by DAMMIN (B). Fit of the theoretical scattering curve (red) to the MCR-ALS scattering data for the dimer (C).

The first insights into the full-length structure of the barley SGT1 protein were performed using the *ab initio* shape determination method. The pure scattering function for the monomeric SGT1 protein, taken from the MCR-ALS analysis, was used to obtain the low-resolution structure in solution using the *ab-initio* approach implemented in the DAMMIN program [46]. The packing radius of the dummy atoms was 0.6 nm, and the total number of non-solvent dummy atoms was approximately 5300. During the modeling process, symmetry and shape restrains were not imposed. The final model obtained after averaging 10 independent DAMMIN models is presented in Figure 9. The monomeric form of the barley SGT1 protein has an elongated shape, with a bend in the middle of the molecule. All three structures of barley SGT1 domains can be unambiguously fit into the DAMMIN model (Figure 9). To fit the SGT1 domains into the low-resolution model of the full length SGT1 protein, the regions between these domains must be elongated and must therefore be unfolded or have very little secondary structure content. The sequences of the VR1 and VR2 regions are much shorter in the barley SGT1 than in the folded domains (TPR, CS, SGS), but in our model these regions have nearly the same dimensions as the folded domains. To independently confirm these assumptions, another low-resolution modeling program, GASBOR [47], was used. The GASBOR model (Figure 9) had a very similar geometry, with an elongated shape and a slight bend in the region in which the CS domain is located (i.e., in the middle of our model). The low resolution models from DAMMIN and GASBOR fit very well to the experimental monomeric barley SGT1 scattering curve, as presented in Figure 9. The same methodology was also applied to obtain the low-resolution shape of the barley SGT1 dimer. The low-resolution molecular model of the dimer obtained from DAMMIN has an elongated shape, similar to that observed for the SGT1 monomer. Based on the fit of the three domains of the barley SGT1 protein into the model of the SGT1 dimer, one can conclude that the TPR domains act as a hub with the CS-SGS domains protruding in the opposite directions (Figure 10). The superposition of the SGT1 monomer model obtained from GASBOR with the model of the SGT1 dimer clearly indicates a large dimerization interface.

### Rigid body modeling of the barley SGT1 protein structure

As previously shown, SAXS can help to predict the solution structure of a particle on the basis of *ab initio* methods [22]. Moreover, using crystallographic and NMR structures, or structures obtained on the basis of bioinformatics tools, the macromolecular structure in solution can be proposed using experimental SAXS data and rigid body modeling.

**Figure 11:**
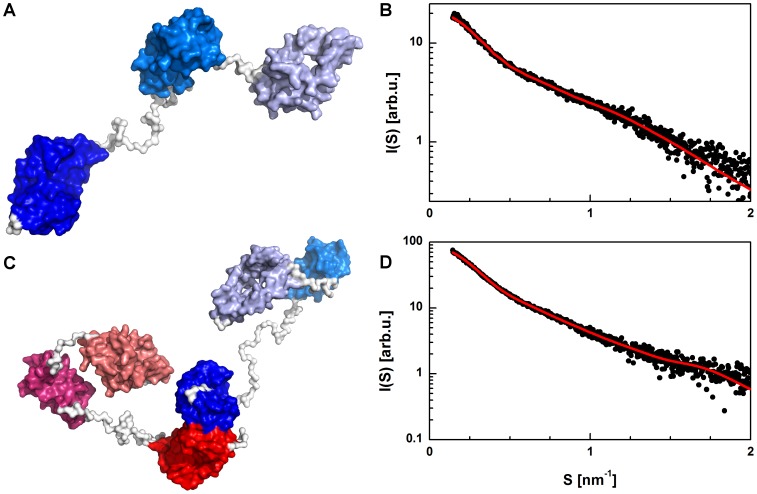
Rigid body modeling of the barley full-length SGT1 protein. Model of the SGT1 monomer obtained using Bunch (A). Fit of the Bunch model to the MCR-ALS monomer scattering data (B). Model of the SGT1 dimer obtained using CORAL (C). Fit of the CORAL model to the MCR-ALS scattering data (D).

To confirm the barley SGT1 models obtained from *ab initio* modeling of low-resolution structures, we also applied rigid body modeling, using the program BUNCH [48] from the ATSAS package. To perform rigid body modeling, we first modeled SGT1 domains using the protein structure prediction servers QUARK and I-TASSER. In rigid body modeling, the flexible regions between the rigid domains were represented as dummy residues with no structural constraints (Figure 11). Additionally, no symmetry constraints were used in the calculations. The resulting model of the barley SGT1 monomer has an extended conformation, with rigid domains behaving similar to beads on a string. The variable regions VR1 and VR2 are disordered and adopt an extended conformation. The extended shape and long unfolded regions give the monomeric barley SGT1 a high degree of flexibility and dynamics, and, surprisingly, they do not cause aggregation. To check whether the dimeric form of SGT1 also adopts an extended and flexible conformation, the barley SGT1 dimer was modeled using the same method as in BUNCH; however, in this case, the method was implemented for multichain proteins in the program CORAL [48]. For this procedure the contacts between the TPR domains (from the SASREF model) were used as constraints to obtain more reliable results, and no symmetry constraints of the SGT1 dimer were assumed. As expected, the barley SGT1 dimer is also highly flexible, and has a dynamic conformation with VR1 and VR2 unfolded. The dimerization only affects the TPR domains, which undergo oligomerization, but it does not influence the other parts of the protein (Figure 11).

**Figure 12:**
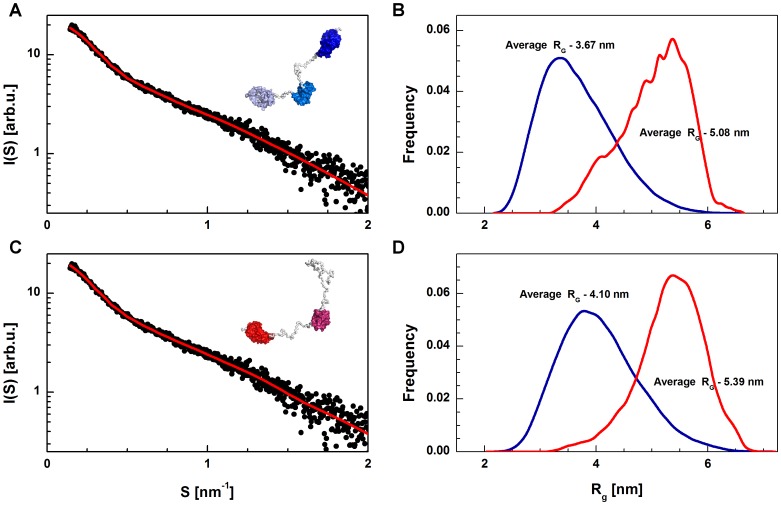
The results of the EOM modeling of the full-length barley SGT1 flexible structure. Models are shown in two ways: by using structural models of all the SGT1 domains (top), and by using models of only the TPR and CS domains (the SGS domain was fixed as unfolded) (bottom). Fits of the best profiles (red) determined by the EOM modeling to SAXS data (A,C). Radii of gyration profile of the starting random pool of structures (blue) and profile of the final optimized ensemble (red) (B,D).

To confirm the results of the rigid body modeling, the ensemble optimization method (EOM) was chosen [21]. This method randomly generates a large number of models of multidomain proteins using the rigid body approach. Then, using a genetic algorithm, the fraction of the models that create an ensemble with the best fit to the experimental data are selected. Unfortunately, the EOM method can be applied only to single-chain proteins, so it was possible to test only the monomeric form of the barley SGT1. The models of the barley SGT1 flexible structure were tested in two ways: by using structural models of all SGT1 domains, and by using models of only the TPR and CS domains (the SGS domain was fixed as unfolded). The rigid body analysis performed for the structural models of all domains agreed well with our experimental data. However, the possibility that the SGS domain has a dynamic character with a minor fraction of secondary structure cannot be excluded. A random pool of the SGT1 monomer models with the folded SGS domain is characterized by an average radius of gyration R_G_ = 3.67 nm, and a pool of models with the SGS domain represented as dummy residues (unfolded domain) is characterized by a R_G_ value of 4.10 nm (Figure 12). After minimization of the final ensemble, the histogram of R_G_ values differs significantly from the histogram generated for the random starting pool.

The model with the folded SGS domain is characterized by an average R_G_ value of 5.08 nm. The model with the unfolded SGS domain is characterized by R_G_ value of 5.39 nm which is slightly higher than the R_G_ value calculated directly from the experimental data using 1 M NaCl. In both the cases, bell-shaped R_G_ histograms were observed, with an additional maximum just above 4 nm for the model with the folded SGS domain. On the basis of analysis of the R_G_ histogram (Figure 12), we concluded that the barley SGT1 monomer has some degree of flexibility but has a limited range. The random pool of the conformations tested (representing all conformations of the particle modeled) is characterized by the fact that the initial R_G_ histogram is significantly different from the final one (refined pool of conformations). This difference suggests that the variable regions (VR1 and VR2) of barley SGT1 provide some flexibility but also ensure the rigidity and extended conformation of the protein. The fit to the extracted scattering data for the monomeric SGT1 model is slightly better for the EOM procedure than for the structure with an unfolded SGS domain (χ^2^ = 1.267), in comparison to the folded SGS domain model (χ^2^ = 1.289). The observed difference is most likely caused by the quality of the SGS domain model generated by I-TASSER.

## Discussion

It is known that the human SGT1 protein does not dimerize under the conditions in which the SGT1 proteins from *Arabidopsis thaliana* and *Saccharomyces cerevisiae* form dimers [19]. A comparison of the amino acid sequences of SGT1 proteins from various species has shown that the region of plant SGT1 that contains charged or polar residues participates in dimer interface formation. In contrast, such polar residues are not found in the human SGT1 protein. In the polar region (in the vicinity of Glu15) of the human SGT1 protein, there is a phenylalanine residue and other residues that are not conserved. At the end of the TPR domain, there are two charged residues (glutamic acids in plants or lysines in yeast) that are absent in the human SGT1 protein: the first charged residue is substituted in the human homolog by glycine, and the second by serine. These sequence substitutions could destabilize the C-terminal part of the TPR domain and prevent dimerization of the human SGT1. A mutagenesis study conducted on AtSGT1b identified only one mutation in the TPR domain, Glu119Gly, which results in a dominant-negative phenotype in the immune response to the PXV virus, mediated by the NB-LRR receptor Rx in *Nicotiana benthamiana* [9]. The dimerization of the SGT1 protein is most likely functionally important, but its role in plants has to be experimentally proven.

In the current study, the structure of the full-length barley SGT1 protein in solution has been determined. As can be expected from a flexible multidomain protein, all three structural domains behave independently. Similar observations were made when the results of the NMR structural studies of the isolated domains and the full-length human SGT1 protein were compared [11]. Although the exact molecular mechanism of SGT1's action is not known, many biochemical studies have revealed that SGT1 most likely interacts with other proteins by linking different protein complexes. For example, the SGT1 protein interacts with ubiquitin ligase complexes and with heat shock proteins [2], [9], [11], [12], [18]. Through dimerization, the SGT1 protein could provide a functional connection between protein degradation and chaperone stabilization of the inactive/active conformation of protein complexes (for example the Rx NB-LRR receptor complex [16]). Using molecular docking and modeling techniques, we predict a possible dimerization model of the barley SGT1 TPR domains. Although structures of TPR dimers, even with a similar closed topology, have been reported, they all have a hydrophobic rather than a polar or charged interface. The polar or charged character of the interface can result in possible changes in structure in response to changes in ionic strength in the cytoplasm or the nucleus. It can therefore shift the equilibrium between the monomer and the dimer and have an impact on SGT1 protein function under stress conditions. The dimerization process of the yeast SGT1 protein is essential, especially for kinetochore assembly [18]. However, there may be another role for the dimerization process. TPR domains are known to bind peptides on the inner concave surface [54]; blocking the inner surface by dimerization may be an as yet unknown method to regulate TPR domain functions.

It was possible to extract the pure scattering function of monomeric and dimeric species from the monomer-dimer mixture using SVD and MCR-ALS analysis. This allowed the modeling of a low-resolution structure. The barley SGT1 monomer, in solution, has an elongated shape with a slight bend in the middle, with the domains behaving similar to rigid beads on a string. The bend occurs at the site at which the CS domain is located. The dimeric form of the barley SGT1 has a similar elongated shape. It was possible to fit all three models of SGT1's domains into our low-resolution model of the dimer. The TPR domains form a hub from which the rest of the protein protrudes in opposite directions. Both ends of the SGT1 protein can help bring molecular complexes together to perform special tasks inside the cell. As shown by rigid body modeling, the regions between TPR, CS and SGS domains are natively unfolded and have a stretched conformation that confers flexibility and dynamics upon the barley SGT1 protein. Additionally, on the basis of our CD experiments, we can assume that all 84 amino acids of SGS are rather unfolded; 22% of secondary structure content should be in the random coil conformation in comparison to the estimated 12%. Therefore, the SGS domain studied may not be fully unfolded. SGS domain is the most conserved part of SGT1 proteins. The second class of proteins with SGS domain are Calcyclin-binding proteins (CacyBP) [55], [56] to which C-terminal fragment of barley SGT1 is similar (33 amino acid fragment: 312–342). Calcyclin-binding proteins are involved in calcium dependent protein degradation by the interactions with ubiquitination machinery proteins like F-box protein Skp1 and E3 ubiquitin ligase SIAH1 [57]. One of the important proteins determining the degradation of CacyBP is beta-catenin [57]. CacyBP C-terminal fragment is unfolded in solution and is responsible for S100A6 (calcyclin) interaction [56]. Up to date, no structures of SGS domain from SGT1 proteins have been determined. Only the structures of SGS domain from CacyBP were determined experimentally. The structure of SGS fragment of human CacyBP/Siah-1 interacting protein - SIP was solved using solution NMR in the complex with S100A6 protein (PDB code: 2JTT) [58]. In this complex SGS fragment adopts alpha helical conformation, consisting of two nearly perpendicular alpha-helices. Also in the NMR structure of Mouse CacyBP protein (PDB code: 1X5M), N-terminal fragment of SGS domain adopts α-helical conformation. This strongly suggests that although its seems that SGS domain is intrinsically unfolded in solution, it can fold upon partner binding or in more crowded, cellular environment.

EOM studies revealed that the best fit to the rigid body model of the barley SGT1 protein was obtained using an ensemble of models with the average radius of gyration equal to 5.10 nm, larger than that for the monomeric form. Dimerization also does not have any impact on the conformation of the whole protein, because only TPR domains participate in this process. It is also possible that the VR regions may play some functional role. The long unfolded region with clustered hydrophobic residues may bind unfolded or hydrophobic regions of other proteins. SGT1, as a protein interacting with HSP90, can also bind other unfolded proteins quite well. Thus, the SGT1 protein can function to load proteins onto HSP90. A similar condition was observed for the p23 protein, which has a 50 aa C-terminal tail that is required for its intrinsic chaperone activity [59]. However, more detailed experiments are needed to better understand a possible role of dimerization and to gain more exact insights into the mechanism of dimerization. It is also very important to search for possible roles of the variable regions of SGT1 to clarify their role in HSP90 chaperone function.

## Supporting Information

Figure S1 Structural alignment of various TPR domains to the barley SGT1 TPR domain model. Hop TRP1A – PDB ID: 1ELW [60] (light pink), Hop TRP2A – PDB ID: 1ELR [60] (grey), design consensus-based TPR oligomer CTPR3Y3 – PDB ID: 2WQH (yellow) [61], design idealized TPR motif PDB ID: 1NA0 (green) [62], barley SGT1 TPR (pink). RMSD and description of used structures are combined in Table S1. The alignment was prepared using Dali structure alignment server (http://ekhidna.biocenter.helsinki.fi/dali_lite/start) [63].
(TIF)

Figure S2 Experimentally determined structures of SGT1 CS domain from *Arabidopsis thaliana* – PDB ID: 2JKI chain S [64] (green), and *Homo sapiens* – PDB ID: 1RL1 chain A [65] (blue) aligned with model of CS domain from *Hordeum vulgare* (pink) used in SAXS modeling of full length SGT1 dimer and monomer. Human and barley SGT1 CS domains possesses 40% sequence identity and 1.0 Å RMSD between aligned structures. *Arabidopsis thaliana* SGT1a CS domain possesses 60% sequence identity and 1.6 Å RMSD between aligned structures. Alignment was prepared using Dali structure alignment server (http://ekhidna.biocenter.helsinki.fi/dali_lite/start) [63].
(TIF)

Figure S3 The experimental CD data (A) for barley CS-SGS protein (red) and CD spectrum obtained for CS-SGS protein in the presence of 30% Ficoll 70 (blue). CD spectra (B) for CS domain from barley SGT1 protein (red) and CS domain of SGT1a from *Arabidopsis thalina* (reference protein) (blue).
(TIF)

Table S1 Structural alignment of various TPR domains to the barley SGT1 TPR domain model.
(DOCX)
