# Supplementary figures and images for: Low-Resolution Structure of the Full-Length Barley (Hordeum vulgare) SGT1 Protein in Solution, Obtained Using Small-Angle X-Ray Scattering
Source: PLoS One. 2014 Apr 8;9(4):e93313. doi: 10.1371/journal.pone.0093313 (PMC3979677; doi:10.1371/journal.pone.0093313)

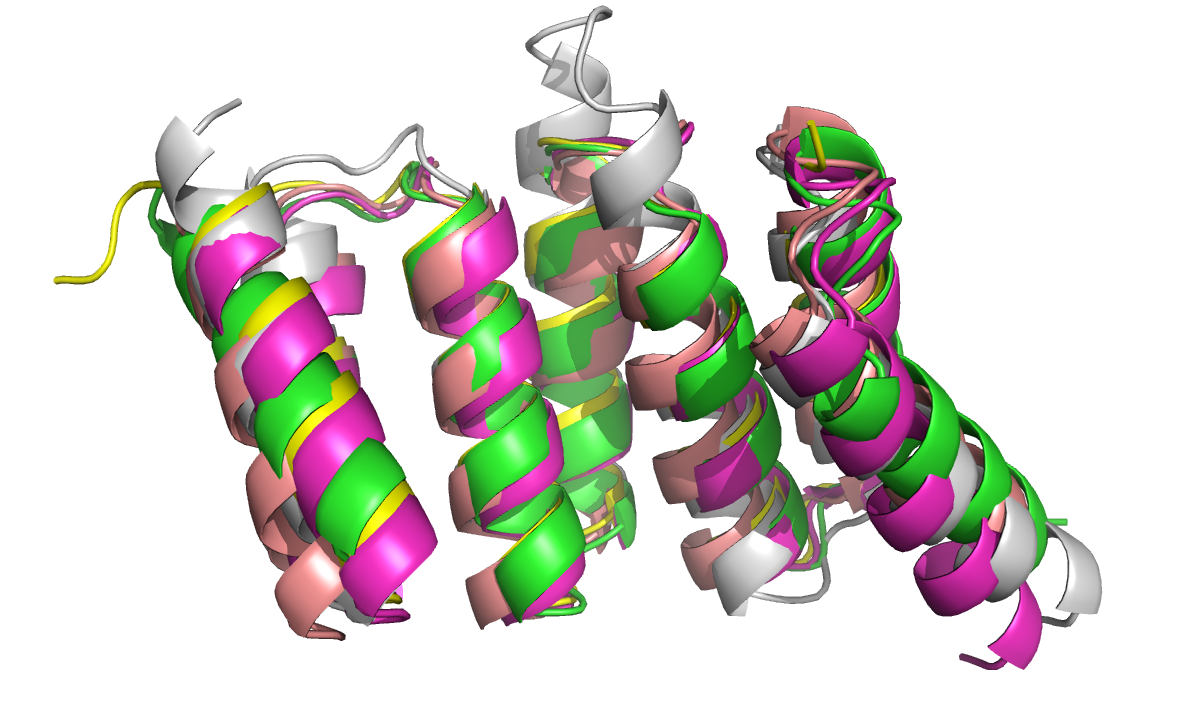

Supplement: Figure S1 — Structural alignment of various TPR domains to the barley SGT1 TPR domain model. Hop TRP1A – PDB ID: 1ELW [60] (light pink), Hop TRP2A – PDB ID: 1ELR [60] (grey), design consensus-based TPR oligomer CTPR3Y3 – PDB ID: 2WQH (yellow) [61], design idealized TPR motif PDB ID: 1NA0 (green) [62], barley SGT1 TPR (pink). RMSD and description of used structures are combined in Table S1. The alignment was prepared using Dali structure alignment server (http://ekhidna.biocenter.helsinki.fi/dali_lite/start) [63]. (TIF) [file pone.0093313.s001.tif]

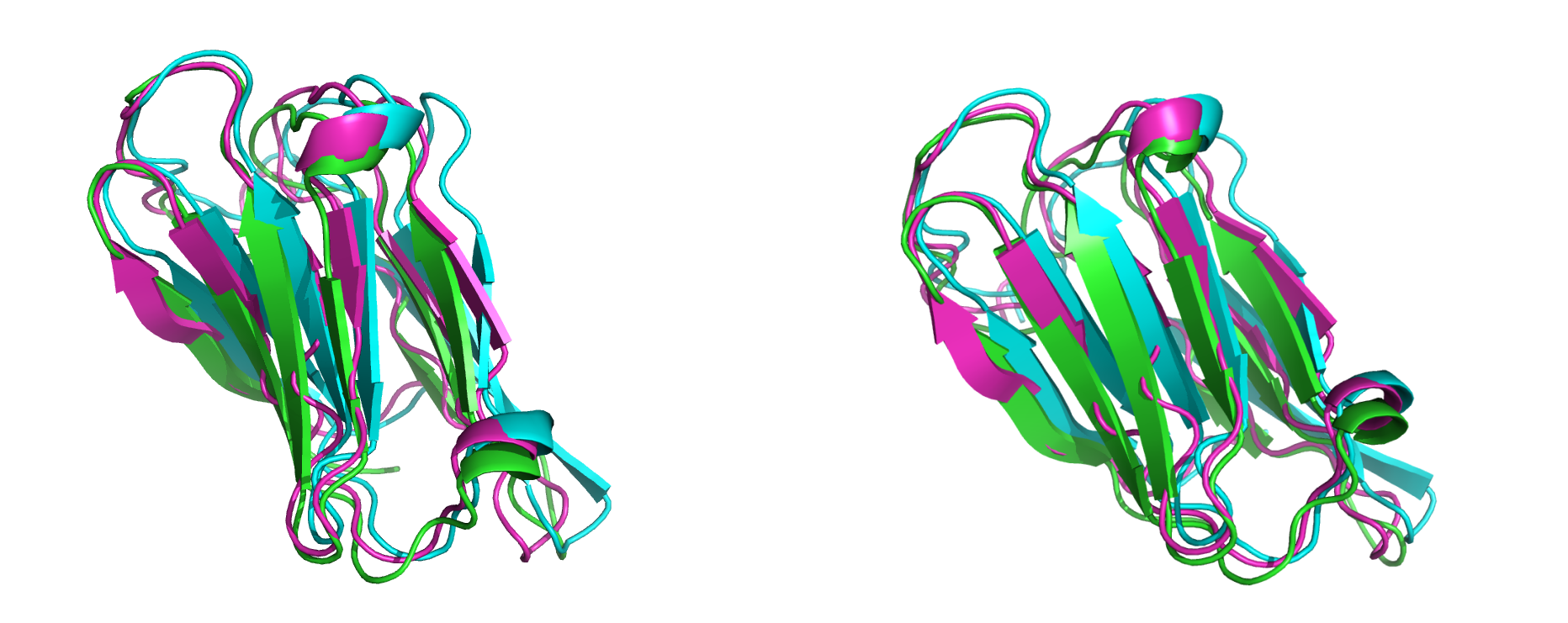

Supplement: Figure S2 — Experimentally determined structures of SGT1 CS domain from Arabidopsis thaliana – PDB ID: 2JKI chain S [64] (green), and Homo sapiens – PDB ID: 1RL1 chain A [65] (blue) aligned with model of CS domain from Hordeum vulgare (pink) used in SAXS modeling of full length SGT1 dimer and monomer. Human and barley SGT1 CS domains possesses 40% sequence identity and 1.0 Å RMSD between aligned structures. Arabidopsis thaliana SGT1a CS domain possesses 60% sequence identity and 1.6 Å RMSD between aligned structures. Alignment was prepared using Dali structure alignment server (http://ekhidna.biocenter.helsinki.fi/dali_lite/start) [63]. (TIF) [file pone.0093313.s002.tif]

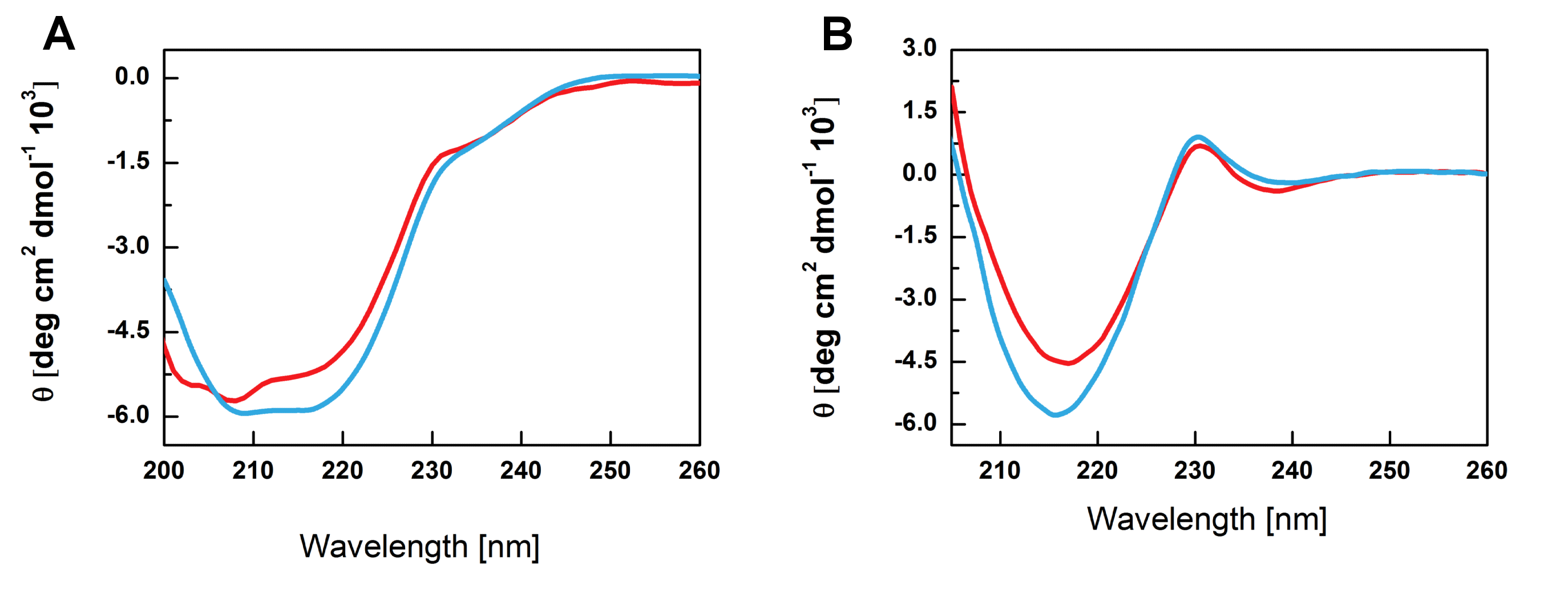

Supplement: Figure S3 — The experimental CD data (A) for barley CS-SGS protein (red) and CD spectrum obtained for CS-SGS protein in the presence of 30% Ficoll 70 (blue). CD spectra (B) for CS domain from barley SGT1 protein (red) and CS domain of SGT1a from Arabidopsis thalina (reference protein) (blue). (TIF) [file pone.0093313.s003.tif]
