# Supplementary material for: Low-Resolution Structure of the Full-Length Barley (Hordeum vulgare) SGT1 Protein in Solution, Obtained Using Small-Angle X-Ray Scattering
Source: PLoS One. 2014 Apr 8;9(4):e93313. doi: 10.1371/journal.pone.0093313 (PMC3979677; doi:10.1371/journal.pone.0093313)
Supplement: Table S1 — Structural alignment of various TPR domains to the barley SGT1 TPR domain model. (DOCX) [file pone.0093313.s004.docx]

**Table S1** Structural alignment of various TPR domains to the barley SGT1 TPR domain model.

| Protein name | PDB ID | Chain | RMSD [Å] | Reference |
| --- | --- | --- | --- | --- |
| Hop TRP1A | 1ELW | A | 1.4 | [60] |
| Hop TPR2A | 1ELR | A | 1.8 | [60] |
| Design consensus-based TPR oligomer CTPR3Y3 | 2WQH | A | 0.8 | [61] |
| Design Idealized TPR motif | 1NA0 | A | 1.1 | [62] |
